# Barriers and enablers to the utilisation of prison-based voluntary medical male circumcision (VMMC) among incarcerated men: a qualitative study from Maula Prison, Malawi

**DOI:** 10.1186/s12889-026-26313-8

**Published:** 2026-01-21

**Authors:** Nelson Tengamowa Munthali, Adrian Musiige, Jim Mtambo, Thokozire Chirambo, Lot Nyirenda

**Affiliations:** 1 Department of Health Systems and Policy, School of Global and Public Health, Kamuzu University of Health Sciences, Private Bag 360, Blantyre, Malawi; 2 Jhpiego Corporation, P.O. Box 1091, Lilongwe, Malawi; 3 Kamuzu University of Health Sciences, Private Bag 360, Blantyre, Malawi; 4 Bwaila Hospital , Lilongwe District Health Office, P.O. Box 1274, Lilongwe, Malawi; 5 Department of Community and Environmental Health, School of Global and Public Health, Kamuzu University of Health Sciences, Private Bag 360, Blantyre, Malawi

**Keywords:** HIV, Voluntary medical male circumcision (VMMC), Prison health, Incarcerated men, Malawi

## Abstract

**Background:**

The prevalence of HIV in Malawi is 7.1% among the general population and 16% among men who are incarcerated, indicating a widespread epidemic. To enhance HIV prevention in the nation, the Ministry of Health has made efforts to offer voluntary medical male circumcision (VMMC) services in prisons as an intervention to strengthen HIV prevention in the country. However, little is known about the barriers and enablers to the utilisation of VMMC among men who are incarcerated.

**Methods:**

This phenomenological qualitative study aimed to explore the experiences of men who were incarcerated while utilising prison-based VMMC services at Maula Prison in Lilongwe. Data were collected through interviews with 7 men who are incarcerated and 7 prison staff, resulting in a sample of 14 participants. A thematic content analysis approach was used to analyse data from both men who were incarcerated and prison staff, guided by the social ecological model, to identify individual, interpersonal, and organisational factors influencing VMMC utilisation.

**Results:**

Both enablers and barriers to VMMC utilisation were identified. Key enablers included improved hygiene, motivation from peers and prison staff, availability of peer educators in the prison, exemption from hard labour during recovery and the availability of VMMC services within the prison. Major barriers consisted of fear of post-operative pain and complications, stigma and mockery from peers, spread of misconceptions about VMMC, shortages of healthcare personnel, limited VMMC-specific training among healthcare workers, overcrowded prison cells, and inadequate post-operative support resources such as soap, water, and nutritious food.

**Conclusion:**

The study concluded that while VMMC is generally acceptable among men who are incarcerated, utilisation is shaped by a combination of individual knowledge, interpersonal influences, and organisational conditions within the prison environment. Addressing these multilevel barriers is essential to enhancing VMMC utilisation among men who are incarcerated.

## Introduction

Human Immunodeficiency Virus (HIV) remains a major global health concern, despite progress in prevention and treatment, with an estimated 40.8 million people living with HIV globally [1]. Africa has been the most highly impacted region, with 26.5 million people living with HIV in 2024 [2]. Malawi, a low-income country in sub-Saharan Africa, has one of the highest HIV prevalence among adults [3]. According to the World Bank open data and the National AIDS Commission (NAC) in 2023, the HIV prevalence among adults aged 15 years and older in Malawi stood at 7.1%, with at least one million people living with HIV (PLHIV) in Malawi [4, 5].

Globally, HIV prevalence among men who are incarcerated tends to be much higher than in the general population [6, 7]. A systematic review and meta-analysis of the global view of HIV Prevalence in Prisons showed that HIV prevalence was highest in Africa [8]. The East and Southern African regions report some of the highest HIV prevalence among men who are incarcerated, with figures reaching as high as 41% in South Africa, 28% in Côte d’Ivoire, and 27% in Zambia [9].

Given the high burden of HIV in prison facilities, the WHO and national governments have emphasised the need to integrate VMMC into comprehensive prison-based health services. VMMC is a surgical procedure that involves removing the foreskin of the penis and has been shown to reduce the risk of acquiring HIV, sexually transmitted infections (STIs), and urinary tract infections [10–15]. As a result, VMMC has been widely promoted as an effective HIV prevention strategy [4, 5, 10]. Countries such as Namibia and Botswana have already implemented structured VMMC programs within correctional facilities, demonstrating the feasibility of such interventions and their potential contribution to broader public health goals [8, 16].

In Malawi, the Ministry of Health (MoH) has made efforts to provide prison-based VMMC services in major prison facilities [17]. These services are offered to incarcerated men as one of the strategies aimed at enhancing HIV prevention [17]. Despite the implementation of these strategies, HIV prevalence remains at 16% among men who are incarcerated, with prevalence as high as 27% in central prisons [9]. There is limited understanding regarding barriers and facilitators to utilisation of prison-based VMMC among men who are incarcerated in Malawi’s prisons, as there is a scarcity of studies that have comprehensively explored these issues. This limited understanding, combined with the high HIV burden in Malawian prisons, justifies the need for this study to address the existing gap to provide an in-depth understanding of the issues being investigated. This understanding will enable the implementation of strategies that may enhance utilisation of VMMC services among men who are incarcerated.

## Methodology

### Research design

This study used a qualitative approach and employed a phenomenological study design, which aims to gain an in-depth understanding of a phenomenon and explores the essence, meaning and significance of the experience from the perspective of the individual who has lived it [18, 19]. The phenomenological design aims at obtaining a deep understanding of the lived experiences of the people [20]. This design was appropriate as it obtained a detailed understanding of the lived experiences and perceptions in regards to utilisation of VMMC services.

### Conceptual framework – social ecological model

**Fig. 1:**
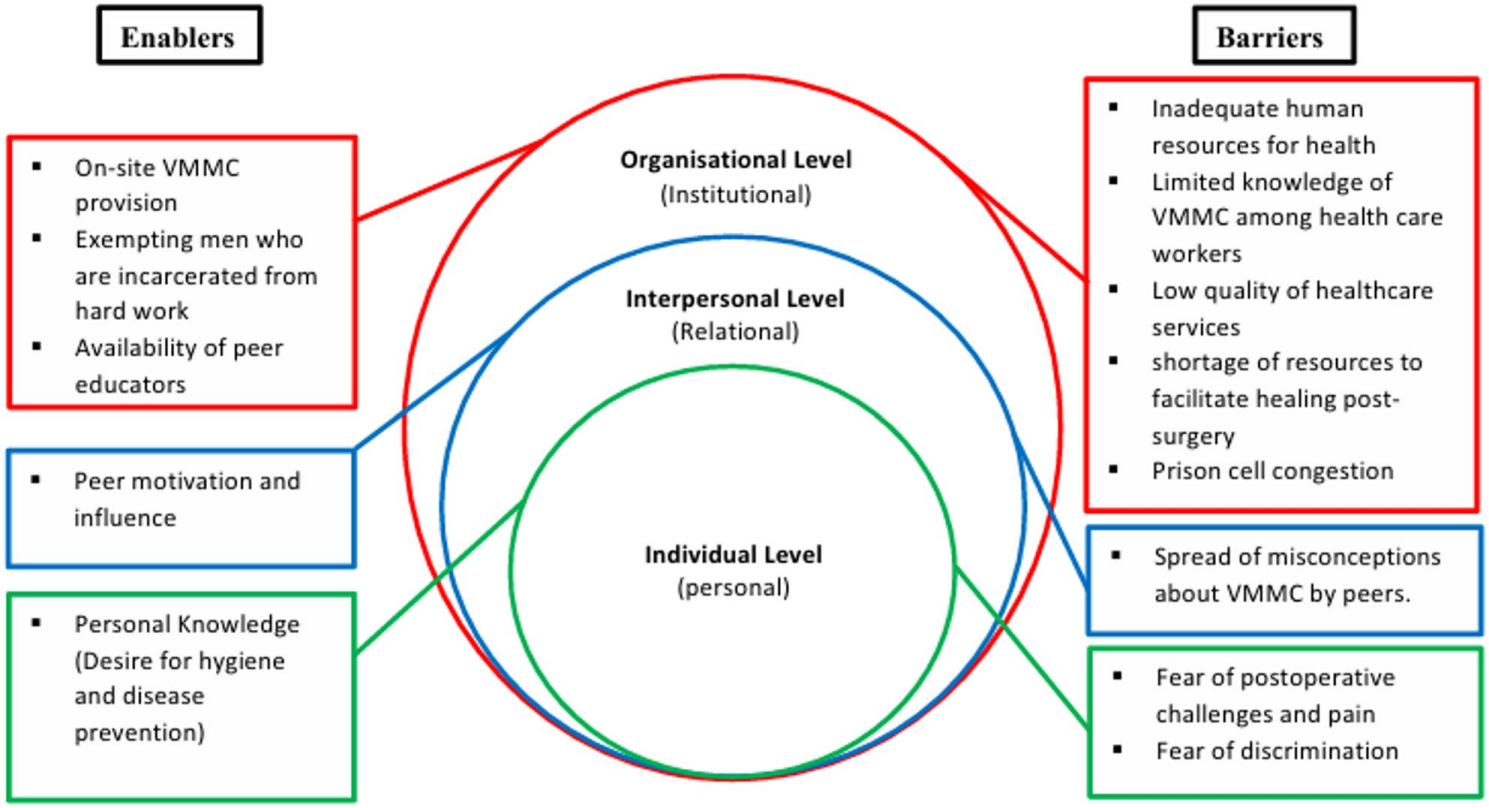
Adapted social-ecological model tailored to barriers and enablers of prison-based VMMC utilisation among incarcerated men at Maula Prison, Malawi

The social-ecological model (SEM) was used as a conceptual framework for this study (Fig. 1). The SEM is vital for understanding complex issues such as experiences in accessing healthcare, as it helps to visualise individual health within its broad contexts [19]. Health inequalities cannot be understood independently of social dynamics and organisational decisions that shape access to healthcare [21, 22].

The SEM used in this study categorised context into three levels: individual, interpersonal, and organisational. These model levels demonstrate the interconnectedness of multiple levels of influence in access to healthcare and provide a platform for multilevel interventions that would effectively improve access to HIV healthcare services for the study population [23]. Therefore, using this model provides a better understanding of the interactions of individual, interpersonal, and organisational level factors, especially the prison healthcare services and prison environment factors, and how their interactions affect the utilisation of the prison-based VMMC program [24].

### Study setting

The study was conducted at Maula prison in Lilongwe, located in the central region of Malawi. It is one of the central prisons where the prison-based VMMC program was implemented in 2023. Above all, Maula prison has the largest population of men who are incarcerated compared to other prisons in Malawi. As of 2025, there were at least 3,278 men who were incarcerated at Maula Prison [25].

### Participants and eligibility criteria

The study involved men who were incarcerated and prison staff aged 18 years and over who were serving prison sentences and working at Maula Prison, respectively, and who were willing to participate.

For men who were incarcerated, eligibility was limited to those who had been reached with VMMC health promotion messages through the prison-based VMMC program, regardless of their circumcision status. Those on remand were excluded due to their legal status and the potential for interruptions in the study process caused by ongoing court proceedings. In addition, those with cognitive impairments or medical issues that could limit meaningful participation were excluded, as were those held in isolation.

For prison staff, the study included health personnel involved in the delivery of healthcare services, health promotion, or management of VMMC services at Maula Prison. The staff targeted were only those involved in healthcare delivery, such that they offered an understanding of organisational issues that may influence utilisation of VMMC, which men who were incarcerated may not fully observe or articulate due to power imbalances. Staff members unavailable during the data collection period were excluded.

### Sample selection

The SEM framework guided the selection of diverse participant perspectives, which included men who were incarcerated and staff, to capture data across all levels. The purposive sampling method was used to recruit prison staff. The researcher selected the staff involved in providing various forms of healthcare support, such as VMMC services [26]. The staff sample included 2 nurses, 2 clinical officers, 1 Environmental Officer, 1 Nutritional Officer, and 1 Pharmacist. Snowball sampling was used to recruit the men who were incarcerated, as other participants, as well as peer educators, recommended other men who were incarcerated due to their understanding as well as knowledge of the subject matter. The sample size was determined by information need, as upon collecting data from 7 men who were incarcerated and 7 staff, rich data were obtained to address the research question; no new themes emerged despite diverse perspectives (e.g., circumcised versus uncircumcised men who were incarcerated). This was determined through iterative analysis during data collection, where thematic redundancy was observed, ensuring rich, comprehensive insights aligned with the study’s phenomenological focus on depth rather than breadth.

### Data collection

The data were collected and analysed between January 2024 and June 2024. The study used interview guides to collect data through in-depth interviews (IDIs), which had open-ended and non-directive questions to demonstrate phenomenology in practice. Interview questions were structured around the SEM’s levels to ensure comprehensive exploration of individual beliefs, peer influences, and organisational barriers to VMMC utilisation. The first author collected data. The tool was made up of demographic data questions and unstructured questions regarding the individual, interpersonal, and organisational factors that influence the utilisation of prison-based VMMC programs among men who are incarcerated and potential solutions to improve the utilisation of prison-based VMMC services at Maula Prison. The interview guides were developed in English and translated into Chichewa for easy communication and interaction. The interviews were conducted in a private room that was secured at the prison facility. Only the researcher and participant were available in the room, such that confidentiality, voluntary participation, as well as privacy were enhanced. Interviews were audio-recorded with participant consent, obtained through written and verbal forms (accommodating literacy levels). Participants were informed that recordings excluded identifiable information. To protect anonymity, recordings were made on a password-protected digital recorder and transferred immediately to a protected computer server accessible only to the lead author.

### Data analysis

The study used a thematic data analysis technique. The analysis began with a holistic reading of transcripts to grasp the whole experience, followed by identifying meaning units, where themes were identified. The first phase involved familiarisation with the recorded interviews to gain an in-depth understanding of the material. This was followed by a verbatim transcription of the recordings. The Chichewa interviews were transcribed and then translated into English to help with data processing and reporting. The translation was conducted by an experienced research assistant, fluent in both Chichewa and English. This individual was not part of the core research team; however, he received training from the lead author on the objectives of the study and the importance of preserving participants’ intended meanings. A second translator performed translation on a few selected sample recordings to minimise bias. The next phase was coding, which involved categorising data and highlighting words, sentences, and phrases relevant to the study topic and objectives. Finally, codes with comparable meanings and properties were grouped into a single category. These categories were utilised to create themes. Sub-themes were identified under each main theme to provide more insights that are refined. As such, a hybrid approach was used, starting with deductively derived main themes and incorporating inductively generated sub-themes that arose during the process of data familiarisation and coding.

## Results

### Participant characteristics

Fourteen individuals participated in the in-depth interviews, including 7 men who are incarcerated and 7 prison staff. The age of the participants ranged from 25 to 52 years, with a mean age of 37.8. Table 1 describes the demographic characteristics of the staff participants, while Table 2 presents the demographic characteristics of men who are incarcerated participants.

**Table 1:** Demographic characteristics of the staff participants

Participant ID	Age	Marital status	Education level
Staff KII S	41	Married	Certificate
Staff KII M	28	Married	Diploma
Staff KII H	33	Married	Diploma
Staff KII F	52	Married	Diploma
Staff KII Y	31	Married	Bachelor’s Degree
Staff KII D	40	Married	Diploma
Staff KII A	38	Married	Diploma

**Table 2:** Demographic characteristics of men who are incarcerated

Participant ID	Age	Circumcision status	Education level	Length of sentence
Inmate KII P	44	Circumcised	Secondary	16 years
Inmate KII E	43	Uncircumcised	Secondary	5 years
Inmate KII C	25	Circumcised	Secondary	12 years
Inmate KII B	36	Uncircumcised	Secondary	14 years
Inmate KII Z	44	Uncircumcised	Tertiary	10 years
Inmate KII X	27	Circumcised	Tertiary	12 years
Inmate KII Q	47	Circumcised	Tertiary	10 years

### Presentation of findings by themes

The findings of this study highlight how individual, interpersonal, and organisational factors collectively influence decisions to utilise prison-based VMMC services among men who are incarcerated. In this study, “peers” refers to fellow incarcerated men, while “peer educators” refers to incarcerated individuals formally identified and trained under the prison health system.

### Theme one: enabling individual factors for utilisation of VMMC

Personal knowledge of the significance of VMMC was identified as an enabling individual-level factor influencing its utilisation among incarcerated men.

### Sub-theme one: personal knowledge of the significance of VMMC

Participants reported that their decision to undergo VMMC was influenced by their personal knowledge of its health benefits. The participants had knowledge that VMMC helps in disease prevention and promotes hygiene, hence opting to undergo the procedure. An incarcerated participant explained:

*“I heard that VMMC helps to reduce the risks of contracting STIs as well as transmission of cervical cancer to women. This tells us that when we go out of prison circumcised, we may protect our spouses from developing cervical cancer. And that knowledge made me seek VMMC. (Inmate KII C)” *

Staff participant highlighted:

*“VMMC improves hygiene because when a person is circumcised*,* the foreskin*,* which keeps dirty substances/particles*,* is removed*,* and the surface becomes dry. Others take action based on this knowledge. (Staff KII D)” *

### Theme two: individual barriers to undergoing VMMC

While there are strong motivations for utilising VMMC services, two individual barriers that hinder the decision-making process were identified: fear of post-procedure challenges and peer discrimination.

### Sub-theme one: being afraid of post-procedure challenges

The study found that some men who are incarcerated resist undergoing the VMMC procedure due to some common challenges after the procedure. Some of the challenges the men who are incarcerated were afraid of included post-operative bleeding, pain during and after the procedure, and other challenges, as summarised by one circumcised inmate participant below:

*“The procedure for VMMC is painful*,* which can even last for some days after the procedure. I have been running away from undergoing the procedure for a long time because I observed the painful experience of fellow men who are incarcerated after the procedure*. *I have a friend here who told me that he will never accept the circumcision because he doesn’t want to experience pain after the procedure. (Inmate KII Q)”*

### Sub-theme two: being afraid of discrimination and mockery from peers

The interviews with participants found that many men who are incarcerated were hesitant to undergo VMMC due to fear of discrimination from peers. Some men who were incarcerated were mocking those considering undergoing VMMC, joking that they were being converted to Islam or teasing older individuals who opted for the procedure. This created a barrier to participation for some. A participant summarised the sentiments as follows:

*“Some men who are incarcerated were making fun of each other when somebody wanted to go or had undergone the circumcision. This fear of ridicule or mockery discouraged some men from seeking VMMC services. Stigma is there due to a lack of awareness, but with enough sensitisation before circumcision services, I don’t think we can have people with such views. (Staff KII M)”*

Although classified as an individual-level barrier, fear of mockery is informed by interpersonal experiences - often observed - of peer ridicule, underscoring the cross-level nature of some barriers within the SEM.

### Theme three: enabling interpersonal factors for the utilisation of VMMC

The study identified one interpersonal factor as an enabler to VMMC utilisation: motivation from peers and prison staff.

#### Sub-theme one: being motivated by peers and prison staff

The majority of participants highlighted that men who are incarcerated were motivated to accept VMMC by peers and prison staff, through shared information, that VMMC enhances their hygiene and has a disease prevention benefit. This understanding influenced their decision to undergo VMMC to maintain their personal hygiene and prevent diseases. A man who is incarcerated narrated:

*“Most men who are incarcerated get motivation from their peers, knowing that once they undergo VMMC, they may be protected from contracting STIs, especially when they are released, hence a decision to undergo VMMC. (Inmate KII Q)”*

Another participant added,

*“For example, my cellmate has been circumcised through VMMC for some time now, and due to the good experience he has had over time, he kept encouraging me to undergo VMMC due to hygiene issues from penile odours I used to have; hence, I chose VMMC because it improves hygiene, which was important to me in the prison setting. (Inmate KII P)”*

### Theme four: interpersonal barriers to utilisation of VMMC

The study identified one sub-theme under interpersonal barriers to VMMC utilisation, namely: spread of misconceptions about VMMC by peers.

### Sub-theme one: spread of misconceptions about VMMC by peers

Spread of misconceptions creates social pressure that discourages men who are incarcerated from utilising VMMC services in prison. One participant narrated:

*“Some men who are incarcerated kept telling me bad things about circumcision*,* saying it will affect my sexual pleasure. Some say I won’t be able to have children of my own. After hearing this over and over*,* I got scared*,* and I decided not to go for the circumcision. (Inmate KII B - Uncircumcised)”*

### Theme five: organisational factors influencing VMMC utilisation

Organisational factors were defined as features of both the prison healthcare services and the broader prison environment that influenced the utilisation of VMMC among men who are incarcerated. The study identified two sub-themes under this broader theme of organisational factors: organisational enablers and organisational barriers.

### Sub-theme one: organisational enablers of VMMC utilisation

Three key enablers emerged under the sub-theme, which are: the availability of peer educators; the provision of VMMC in a prison setting; and exempting men who are incarcerated from hard work.

#### The availability of peer educators

The study identified the availability of peer educators in the prison setting as a key organisational enabler to the utilisation of VMMC in the prison. The majority of the participants explained that the availability of peer educators in prison cells was serving as an enabler, since peer educators were providing essential information on VMMC to men who are incarcerated and also looking after them during recovery. A man who is incarcerated narrated:

*“It is quite good as we have peer educators*,* these peer educators sensitise men who are incarcerated daily*,* and it has a great impact because men who are incarcerated learn about the importance of VMMC through them*,* which contributes to increased utilisation of these services. (Inmate KII Q)”*

Another participant added:

*“Every cell has a trained peer educator. Most of the men who are incarcerated look up to these educators for information, and it helps them in their decision-making regarding VMMC as well as wound healing management post-surgery. (Inmate KII X)”*

Support from peer educators and healthcare staff enhanced awareness of VMMC’s protective benefits and positively influenced men’s decisions to utilise the service.

#### The provision of VMMC in a prison setting

Both staff and men who are incarcerated believed that providing VMMC services within the prison was a key factor that enabled men who are incarcerated to undergo the procedure. They indicated that providing these services in prison made it easier for men who are incarcerated to accept and access the procedure. Some participants explained:

*“Some individuals failed to undergo circumcision when they were at home due to various factors such as long distance. The availability of these services here provides the opportunity for one to simply walk to the clinic any day and get circumcised. (Inmate KII E - Uncircumcised)”*

*“The fact that VMMC services are offered right in prison enables men who are incarcerated to easily access the services right in the prison facility without much effort. (Staff KII Y)”*

#### Exempting men who are incarcerated from hard work

The study further found that men who are incarcerated were motivated to undergo VMMC due to measures set aside by the prison authorities. Men who are incarcerated who choose to undergo the VMMC procedure are exempted from hard labour. This exemption from these activities served as a motivator for the men who are incarcerated to participate in the procedure. This highlights the significance of implementing policies and guidelines for the effective implementation of VMMC in these facilities. Some inmate participants, both circumcised, narrated:

*“Every cell has a peer educator*,* and these peer educators take the names of men who want to get circumcised. If a man who is incarcerated was scheduled to go to a farm for work has shown interest in being circumcised*,* he is exempted from going to the field. This motivates men who are incarcerated that*,* after getting circumcised*,* they will be granted some care. (Inmate KII Q)”*

*“Other men who are incarcerated work on farms, as office messengers, and cleaners. However, everyone is allowed to have VMMC. And when they do, they pause their daily work routines until full recovery. (Inmate KII P)”*

### Sub-theme two: organisational barriers to VMMC utilisation

Five key issues emerged under the sub-theme of organisational barriers to VMMC utilisation, which are: inadequate human resources for health; low quality of health care services; limited knowledge of VMMC among prison health care workers; the shortage of resources such as food, soap and water to facilitate healing post-surgery and congestion in the prison cells.

#### Inadequate human resources for health

Both staff and inmate participants expressed concerns about the insufficient number of healthcare workers. They indicated that the current number of healthcare workers involved in providing support to the men who are incarcerated does not meet the healthcare needs of the prison inmate population. One participant explained:

*“The clinic staff are not enough compared to the number of prisoners. Mostly, the clinic is closed because there is no one around. As a result, the provision of VMMC becomes a challenge. (Inmate KII X - Circumcised)”*

Another participant added:

*“Clinic staff are still not enough. As such, they concentrate on providing other forms of healthcare support, such as treatment of other diseases, other than providing VMMC services. This made me delay VMMC. (Inmate KII E - Uncircumcised)”*

#### Low quality of health care services

Inmate participants regarded the quality of healthcare services, overall, to be of low quality, and they believed this prevented some men who are incarcerated from benefiting from VMMC programs. The participants reported that the prison clinic lacks essential drugs that could help in facilitating wound healing. This highlights poor support for the circumcised men who are incarcerated, which may demotivate others to be circumcised. Some participants narrated:

*“Someone does not receive enough medical assistance, especially when one has post-surgical adverse events, as the prison clinic lacks essential drugs for facilitating wound healing and pain management. (Inmate KII P - Circumcised)”*

### Limited knowledge of VMMC among prison health care workers

Both staff and inmate participants expressed their concerns about the limited knowledge of VMMC among healthcare workers at Maula Prison. They explained that these healthcare workers had not received specialised training in VMMC. As a result, they often relied on external healthcare professionals for assistance during emergencies. This is a clear indication that this was leading to delays in patient care, suggesting the need for educational support for the prison staff. Some participants explained:

*“Most of our HCWs inside the prison are not conversant with VMMC services; as such, they do not assist men who are incarcerated when their wounds have complications, such as burst sutures. (Inmate KII P - Circumcised)”*

*“Besides that, we are health care experts; most of us did not receive formal training on VMMC. We just use our own knowledge to support these men who are incarcerated and who develop complications post-surgery. (Staff KII H)”*

#### The shortage of resources such as food, soap and water to facilitate healing post-surgery

The majority of participants expressed concerns about the lack of resources needed by the men who are incarcerated after undergoing the VMMC. The participants were concerned about the lack of soap and nutritional food, which were essential for wound healing. Furthermore, the participants expressed concerns about the scarcity of water in the prison, which prevents them from cleaning the wound. Some participants explained:

*“The biggest challenge is a shortage of nutritional meals offered here in the prison. After receiving VMMC, a high-protein, high-nutrient diet is important for wound healing, which is not offered in this place. Additionally, water challenges affect hygienic practices since we fail to clean up the wounds. (Inmate KII P - Circumcised)”*

*“VMMC is a very good service, but here in prison, there are hygienic challenges. Men who are incarcerated are given one tablet of soap after being circumcised, which is not enough for the wound healing period, and the number of soaps should increase. (Staff KII S)”*

#### Congestion in the prison cells

Congestion in the prison rooms was another barrier they highlighted. This suggests that organisational issues were hindering the utilisation of VMMC services among other men who are incarcerated. One staff participant narrated that:

*“Here in prison*,* we have sickbay rooms reserved for inmates who are ill. Inmates who have been circumcised also stay there during their healing period when there is free space. But sometimes*,* when sickbay rooms are full with inmates with other health conditions*,* the circumcised inmates are sent back to the normal cells*,* which are usually overcrowded with limited space*,* which might affect their wound healing. This makes many men who are incarcerated afraid to undergo VMMC for fear of sleeping in the overcrowded cells if the sickbays are full. (Staff KII A)”*.

## Discussion

The study investigated the barriers and enablers to the utilisation of prison-based VMMC among men who are incarcerated at Maula prison in Lilongwe, Malawi. The social ecological model was used to understand these existing barriers and enablers, with the findings categorised based on individual factors, interpersonal factors, and organisational factors. The enabling individual factor for VMMC utilisation among men who are incarcerated was personal knowledge of its benefits. Study data showed that men who are incarcerated decided to undergo VMMC due to their knowledge of the benefits of being circumcised. Their understanding that VMMC helps to prevent contraction and transmission of some STIs and improve hygiene was influential in their decision to undergo the procedure. This aligns with previous evidence suggesting that adequate knowledge on the benefits of VMMC, such as minimising transmission of STIs, as well as enhancing personal hygiene among men, influenced individuals’ decision to undergo the procedure [27–29].

The study found that fear of post-surgical complications was one of the individual barriers to VMMC among men who were incarcerated. It was found that the men who are incarcerated expressed fear of experiencing pain, bleeding and other post-surgical challenges. This is in line with previous evidence that highlighted that pain, as well as bleeding and other surgical challenges, causes fear among individuals who have never been circumcised, resulting in underutilisation of VMMC [30, 31]. This suggests the need for the provision of information for the men who are incarcerated to understand that pain management is available during the provision of the VMMC procedure, and post-operative care is available to the point that the wound is completely healed.

The current study highlights the complex role of interpersonal factors in influencing VMMC utilisation among men who are incarcerated. While peer and staff support enabled some men who are incarcerated to utilise VMMC, through sharing of information, personal experiences, and motivating others, negative peer influence within the prison environment also acted as a barrier. The spread of misconceptions about VMMC by peers created social pressure that discouraged some men who are incarcerated from utilising the service. These findings are consistent with previous evidence showing that peer and social networks can function both as facilitators and barriers, and that sharing accurate information is a critical strategy for enhancing VMMC utilisation [32].

These study findings indicate that organisational factors within prison settings both enabled and hindered the utilisation of VMMC among men who are incarcerated. Within the Social Ecological Model, these organisational factors reflect features of both prison healthcare services and the broader prison environment that influence VMMC utilisation. It was found that the prison facilities had peer educators, who play a role in supporting various healthcare interventions. Among these interventions was sensitising and educating men who are incarcerated about VMMC. The delivery of VMMC-related information by peers is particularly important in prison settings, where trust in formal healthcare providers may be limited and where incarcerated men may feel more comfortable discussing sensitive issues with individuals who share similar lived experiences. Peer educators may be perceived as more reliable and relatable sources of information, which can enhance understanding, reduce fear, and positively influence decision-making regarding VMMC utilisation. However, reliance on peer-delivered information may also have limitations. Peer educators may unintentionally spread incomplete or inaccurate information in the absence of adequate training and supervision, reinforcing misconceptions and fear surrounding VMMC. This dual role of peer influence aligns with earlier findings in this study, where peers acted as both facilitators and barriers to VMMC utilisation. Previous evidence suggests that peer-led health education can be highly effective when peers are adequately trained, supported, and provided with accurate, standardised information [28, 29].

Beyond healthcare delivery, organisational features of the prison environment also influenced VMMC utilisation. The availability of VMMC services within the prison itself facilitated access by providing an opportunity to those in need to have easy access, as the men who are incarcerated did not require transport to travel a long distance to access the service at external health facilities. Additionally, supportive prison policies, such as exempting men who had undergone VMMC from hard labour to allow adequate wound healing, created an enabling environment that encouraged service uptake. While unclear, it must be acknowledged that this could have had the unintended effect of some men opting for VMMC to gain exemption from prison labour requirements. Ensuring that VMMC is offered in a way that does not appear coercive is a necessary area for future research and programming efforts. These organisational practices demonstrate how administrative support and prison-level policies can positively influence health-seeking behaviour among incarcerated men.

However, the study also identified several organisational barriers that limited utilisation of VMMC services. A major challenge was the limited healthcare workers within prison facilities, which resulted in limited provision of VMMC services. Limited availability of health workers may hinder efforts aimed at providing comprehensive services, including pre-operative counselling, post-operative monitoring, and effective pain management. These gaps undermine trust in men who are incarcerated in the system and discourage others from participating in the program [33]. Furthermore, the facility has limited resources, such as drugs. Men who are incarcerated require painkillers after undergoing VMMC to minimise post-surgical pain. Limited access to painkillers may result in poor utilisation of VMMC. This may be attributed to limited funding support for the prison facilities, which hinders the procurement of essential drugs. Additionally, the findings also revealed gaps in VMMC-specific training among prison healthcare workers. Many healthcare providers relied on informal or self-acquired knowledge due to the lack of formal training opportunities. This limited access to up-to-date, standardised VMMC training may affect the quality of care and reduce confidence among men who are incarcerated in seeking VMMC services.

Conversely, organisational resource constraints within the prison environment acted as significant barriers to VMMC utilisation. Participants reported shortages of essential resources such as soap, water, and nutritious food, all of which are critical for post-operative wound care and recovery. Inadequate access to water and hygiene supplies limited the ability of men who were circumcised to maintain wound cleanliness and prevent infection. Previous studies have similarly shown that lack of essential resources such as soap, water and other hygienic facilities may hinder efforts aimed at enhancing utilisation of VMMC, and evidence has further shown that hygiene is a cornerstone of safe surgical recovery, and its absence in prison settings may undermine the potential health benefits of VMMC [27].

Overcrowding within prison cells further deepened these challenges. Participants described congestion in prison cells, which limited the availability of appropriate spaces for post-circumcision recovery. When sickbay rooms were full, circumcised inmates were often returned to overcrowded cells with limited space, increasing concerns about wound healing and infection. This situation generated fear among men who were incarcerated, discouraging some from opting for VMMC due to uncertainty about post-operative care arrangements. Such findings underscore how structural and environmental constraints within prisons can directly influence health-seeking behaviour and utilisation of preventive health services.

## Limitations of the study

This study was conducted at Maula Prison in Lilongwe, Malawi, and the factors influencing VMMC uptake in this setting may differ from those in other prisons or countries. However, the qualitative design we employed does not seek to achieve generalizability, but rather, transferability, whereby findings from our study setting may be transferable to settings with similar contexts. Given the sensitive nature of the topic and the hierarchical environment of prisons, responses may have been influenced by social desirability bias or fear of repercussions. While efforts were made to ensure privacy and trust, the presence of prison authorities and the researcher’s role may have shaped participants’ openness.

### Practice and research recommendations

Based on the findings, several practice implications are evident:

- Implement targeted awareness initiatives within prison facilities by conducting regular awareness programs to address misconceptions, stigma, and discrimination related to VMMC, including engagement of VMMC program implementers during prison outreach visits.
- Improve resource allocation and infrastructural support for VMMC services by mobilising government and stakeholder resources to address infrastructural gaps, including adequate space and privacy for post-procedure recovery, to ensure sustainable and effective VMMC service delivery.
- Strengthen the capacity of prison healthcare workers for VMMC service delivery through targeted recruitment and standardised initial and refresher training on VMMC procedures, post-operative care, and complication management, supported by ongoing supervision and mentorship to improve quality, continuity, and confidence in prison-based VMMC services.

However, the scope of these practice recommendations and the funding required to enact them may be challenging for their implementation. Furthermore, because the evidence simultaneously suggests that observed enablers were substantial enough to motivate some men to utilise VMMC while observed barriers were substantial enough to constrain others, there is no clear indication on how to prioritise programmatic changes, undermining feasibility. Therefore, future research to investigate how these various factors cooperate to differentially shape VMMC utilisation would be useful. Moreover, research is needed to identify the most influential and modifiable facilitators and barriers or groups of facilitators and barriers, so that resources can be more strategically targeted and intervention programming more efficiently tailored.

## Conclusion

The study demonstrated that men who are incarcerated in Malawi can utilise VMMC and that the utilisation is influenced by a combination of individual knowledge, interpersonal influences and organisational conditions within the prison. However, significant barriers persist, including fear of complications, spread of misconceptions about VMMC by peers, stigma, inadequate health resources, and poor hygiene conditions. While these barriers may be similar to those found among the general population, the prison environment may exacerbate the barriers, thereby necessitating special attention. Such attention is an urgent matter, considering that the prevalence of HIV among men who are incarcerated is higher than that among the general population. Addressing these challenges through targeted education, improved health system capacity, and provision of essential resources is critical to enhancing VMMC utilisation and ensuring safe recovery in prison settings.

## Data Availability

Due to the sensitive nature of the data and to protect participant confidentiality, the interview recordings and transcripts are not publicly available. However, de-identified excerpts relevant to the study findings can be shared upon reasonable request to the corresponding author, subject to ethical approval.

## Abbreviations

AIDS: Acquired Immune Deficiency Syndrome
COMREC: College of Medicine Research and Ethics Committee
HIV: Human Immunodeficiency Virus
MoH: Ministry of Health
NAC: National AIDS Commission
PLHIV: People living with HIV
SEM: Social-ecological model
STI: Sexually Transmitted Infections
VMMC: Voluntary Medical Male Circumcision
WHO: World Health Organisation
